# Impact of *Ureaplasma urealyticum* infection on semen parameters and *in vitro* fertilization outcomes in infertile men

**DOI:** 10.3389/fendo.2024.1484007

**Published:** 2024-11-19

**Authors:** Yangyang Wan, Xin Chen, Zhaoyu Chen, Wenjing Liu, Siyao Li, Juan Hua

**Affiliations:** ^1^ Center for Reproduction and Genetics, The First Affiliated Hospital of USTC, Division of Life Sciences and Medicine, University of Science and Technology of China, Hefei, China; ^2^ Department of Biochemistry and Molecular Biology, School of Basic Medical Sciences, Anhui Medical University, Hefei, China; ^3^ Research Center for Translational Medicine, The Second Hospital of Anhui Medical University, Hefei, China

**Keywords:** *Ureaplasma urealyticum*, male infertility, semen parameters, oxidative stress, neonatal outcomes, *in vitro* fertilization

## Abstract

**Introduction:**

*Ureaplasma urealyticum* (UU) is a common pathogen associated with genital tract infections in infertile males. However, its impact on semen quality, embryo development, and *in vitro* fertilization (IVF) outcomes remains underexplored. This study aims to evaluate the effect of male UU infection on semen parameters, embryo development, pregnancy outcomes, and neonatal health in infertile couples.

**Methods:**

A retrospective analysis was conducted on 1,215 infertile couples at the First Hospital of USTC. Participants were divided into two groups based on the male partner’s UU infection status: UU-positive (n=249) and UU-negative (n=966). Semen parameters (sperm concentration, motility, morphology, anti-sperm antibodies, DNA stainability) were assessed. Embryo development was evaluated through fertilization rates and blastocyst formation rates. Pregnancy outcomes (clinical pregnancy, live birth rates, miscarriage rate) and neonatal health (gestational age, birth weight, Apgar scores, preterm delivery) were also compared.

**Results:**

Semen parameters, including sperm concentration, motility, and morphology, were similar between the UU-positive and UU-negative groups. However, the UU-positive group had significantly higher levels of anti-sperm antibodies (ASA) (*p*=0.020) and higher DNA stainability (HDS) (*p*=0.014). Despite these differences, embryo quality, as measured by fertilization rates and blastocyst formation rates, was not significantly different between the two groups. Pregnancy outcomes, including clinical pregnancy and live birth rates, were also comparable. While the UU-positive group had a slightly higher miscarriage rate, this difference was not statistically significant. Neonatal outcomes, including gestational age, birth weight, Apgar scores, and preterm delivery rate, did not differ significantly between the two groups.

**Discussion:**

The study suggests that while male UU infection may adversely affect certain semen parameters, its impact on IVF outcomes—such as embryo quality, pregnancy rates, and neonatal health—appears to be minimal. These findings support the continued use of IVF as a viable and safe option for infertile couples with male UU infection, as it does not significantly influence reproductive or neonatal outcomes.

## Introduction

Male infertility contributes to nearly 50% of all infertility cases (1), with a wide range of factors influencing male reproductive potential, including infections of the genital tract. *Ureaplasma urealyticum* (UU) is a common pathogen associated with genital tract infections and has been implicated in male infertility (2–4). Although the adverse effects of reproductive tract infections have been well studied in female infertility patients (5), the influence of male UU infections on semen quality and *in vitro* fertilization (IVF) outcomes remain controversial. Some studies suggest that UU infection can negatively affect sperm concentration, motility, and morphology, thereby reducing fertility potential (6, 7). However, other studies have reported that seminal UU infection may not have a substantial negative impact on semen quality, pregnancy rates, or outcomes of assisted reproduction (8).

Some studies have suggested that male UU infection may impair not only semen parameters but also embryo quality and pregnancy outcomes, potentially leading to higher rates of miscarriage and lower live birth rates (9, 10). The pathophysiological mechanisms behind these effects include the induction of inflammatory responses and oxidative stress within the male reproductive tract, resulting in increased sperm DNA fragmentation and decreased nuclear maturity (8). However, other studies have reported conflicting results, showing no significant association between UU infection and adverse reproductive outcomes, suggesting the need for further investigation to clarify the role of UU in male infertility and IVF success (11, 12).

Given the conflicting evidence, this research seeks to thoroughly assess the effect of male UU infection on semen and embryo quality, as well as pregnancy outcomes in infertile couples undergoing IVF treatment. This study aims to provide a clearer understanding of how male UU infection influences IVF reproductive outcomes. The findings could have important implications for the management of male infertility and the optimization of assisted reproductive technology (ART) protocols to improve the chances of successful conception and healthy live births.

## Patients and methods

### Patients

A total of 1,215 infertile couples who underwent IVF cycles at the First Hospital of USTC were enrolled in this study between January 2020 and June 2023. The inclusion criteria for this study were as follows: female factor infertility (such as tubal infertility, endometriosis, ovulatory disorders, and cervical factor infertility), certain male factors (such as mild to moderate oligoasthenoteratozoospermia), and unexplained infertility (with normal ovarian function assessment, tubal patency evaluation, and semen analysis). The 1,215 infertile couples were divided into two groups based on the male partner’s UU infection status: UU-positive (n=249) and UU-negative (n=966). Female participants were screened and excluded based on the presence of UU, *Mycoplasma hominis*, *Chlamydia trachomatis*, *Neisseria gonorrhoeae*, fungal infections, and common gynecological inflammatory markers. Male participants were screened for and excluded based on the presence of UU, *Mycoplasma hominis*, *Chlamydia trachomatis*, *Neisseria gonorrhoeae*, and fungal infections. In the UU-positive group, most male patients were asymptomatic, while a minority experienced mild discomfort associated with urination or ejaculation.

### Semen parameters

Semen samples were collected from patients following a period of 2–7 days of abstinence by masturbation into sterile containers. Standard semen parameters, including sperm volume, concentration, and percentage of spermatozoa with forward motility (PR) and total motility (PR+NP), were assessed in a specialized seminal laboratory using computer-assisted sperm analysis (CASA) (SAS Medical, Beijing, China). The assessment of sperm concentration and motility was carried out by the Saes SAS-II sperm quality analyzer (SAS Medical, Beijing, China). Sperm morphology, leukocyte count, and anti-sperm antibodies were determined through Diff-Quick staining, benzidine peroxidase staining, and mixed antiglobulin reaction, respectively (Anke Biotechnology, Hefei, China). The morphological parameters of sperm and peroxidase-positive cells were analyzed under a microscope (LEICA DM2500, Wetzlar, Germany). All analyses were conducted and evaluated following the World Health Organization (WHO) manual (5th edition). Fluorescence signals from spermatozoa stained with acridine orange were analyzed using the BD Accuri C6 flow cytometer (BD, San Jose, USA). The resulting data were processed using the sperm DNA fragmentation index (DFI) viewer software (Cellpro, Ningbo, China), yielding values for high DNA stainability (HDS) and sperm DFI. The HDS value reflects the percentage of immature nuclear spermatozoa, while the DFI represents the extent of damage to sperm nuclear chromatin.

### Semen STD detection

All semen samples were subjected to UU culture and drug sensitivity testing following the guidelines provided by the manufacturer (Zhong Ai Sheng Hebei Biotechnology Co. Ltd., Xingtai, China). The semen specimens were inoculated into a liquid medium and subsequently distributed into wells containing indicators, such as urea and arginine, to detect the presence of potential pathogens. The culture plates were incubated at a controlled temperature of 36 ± 1°C. UU presence was determined by observing a color change in the medium, which occurs when UU metabolizes urea, leading to an increase in pH and a shift in the medium’s color from orange-yellow to red after 24 hours of incubation. For positive samples, further identification and quantification were performed, with 10^4^ colony-forming units (CFU) per milliliter or higher being considered a positive result. To identify co-infections with other microorganisms, standard laboratory procedures were employed. *Mycoplasma hominis*, *Chlamydia trachomatis*, *Neisseria gonorrhoeae*, and various fungi were screened using specific diagnostic methods. *Chlamydia trachomatis* was detected using latex immunochromatography assays (Abogen Biosciences Co. Ltd., Hangzhou, China). For *Neisseria gonorrhoeae*, standard culturing techniques were applied (Autobio Biosciences Co. Ltd., Zhengzhou, China). Fungal infections were identified through fungal slide culture methods (Tianda Diagnostic Reagents Co., LTD, Hefei, China).

### IVF procedure and pregnancy follow-up

Female partners underwent controlled ovarian stimulation (COS) treatment. Each female was administered 8000 IU of recombinant hCG (Guangdong Lizhu Group Lippo Biochemicals Co., Ltd.) or 250 µg of Azer (Gonapeptyl, Ferring Pharmaceuticals) subcutaneously on the same day when more than two dominant follicles measured ≥ 18 mm in diameter. Eggs were retrieved 34–36 hours later. Fertilization assessment was conducted 16–20 hours post-retrieval, followed by a 24-hour observation period for cleavage and embryo scoring. One to two high-quality embryos were selected for intrauterine transfer on day 3 after retrieval, while blastocyst transfer involved the selection of one blastocyst for transfer on day 5. Elective transfers were performed using frozen embryos. Blood and urinary hCG levels were measured 12–14 days post-embryo transfer. In the event of a biochemical pregnancy diagnosis, clinical confirmation was obtained at 30 days via ultrasound examination, which revealed the presence of a gestational sac, germ bud, and fetal heartbeat *in utero*. Subsequent monitoring included the early, mid, and late stages of pregnancy as well as delivery outcomes and newborn health status.

### Statistical analysis

Statistical analyses were performed using R version 4.3.0 (R Core Team, Vienna, Austria). Continuous variables following a normal distribution were represented as mean ± standard deviation (SD), while those not normally distributed were reported as median (interquartile range, IQR). Categorical variables were represented as frequency (percentage). Student’s t-test was applied for normally distributed continuous variables, whereas the Mann-Whitney U test was employed for non-normally distributed parameters. Pearson’s chi-square or Fisher’s exact test was used for qualitative data analysis. A p-value < 0.05 was considered statistically significant.

## Results

### Baseline characteristics

The analysis of baseline demographic and clinical characteristics in this study’s population, comprising 1,215 male patients from infertile couples, divided into UU-positive (n=249) and UU-negative (n=966) groups. There were no significant differences between the two groups in terms of age, body mass index (BMI), duration of infertility, and infertility types (all *p*>0.05). Additionally, there were no significant differences between the two groups in terms of basal follicle-stimulating hormone (FSH) levels in the female partners (*p*=0.235), endometrial thickness on hCG days (*p*=0.423), and the number of days taken for ovulation induction (*p*=0.321). In summary, the two groups did not differ statistically regarding general clinical data parameters (**Table 1**).

**Table 1:** The general clinical data characteristics of the two groups.

Variables	Total (n=1215)	UU-negative (n=966)	UU-positive (n=249)	*p*
Male age (years; mean ± SD)	32.25 ± 5.04	32.22 ± 5.09	32.38 ± 4.88	0.651
Female age (years; mean ± SD)	33.60 ± 5.88	33.60 ± 5.82	33.57 ± 6.12	0.950
BMI of male (kg/m^2^; mean ± SD)	24.80 ± 3.39	24.74 ± 3.22	25.05 ± 3.98	0.254
BMI of female (kg/m^2^; mean ± SD)	22.75 ± 3.27	22.83 ± 3.29	22.45 ± 3.18	0.102
Duration of infertility (years; mean ± SD)	2.91 ± 2.34	2.89 ± 2.28	3.00 ± 2.59	0.529
Types of infertility				0.665
Primary infertility (n (%))	532 (43.79)	426 (44.10)	106 (42.57)	
Secondary infertility (n (%))	683 (56.21)	540 (55.90)	143 (57.43)	
Female basic FSH (mIU/ml; mean ± SD)	7.56 ± 2.63	7.51 ± 2.56	7.75 ± 2.87	0.235
Female endometrial thickness (mm; mean ± SD)	10.54 ± 2.64	10.57 ± 2.70	10.42 ± 2.42	0.423
Ovulation induction (days; mean ± SD)	9.63 ± 4.16	9.57 ± 4.13	9.87 ± 4.31	0.321

UU, *Ureaplasma urealyticum*; SD, standard deviation; BMI, body mass index; FSH, follicle stimulating hormone.

### Semen parameters

The comparison of semen parameters between the UU-positive and UU-negative groups of male infertile patients showed no significant differences in abstinence time (*p*=0.744) or semen volume (*p*=0.566). Sperm concentration was similar between the two groups (*p*=0.706). The median progressive motility was marginally greater in the UU-positive group (41.77%) compared to the UU-negative group (39.22%), though this difference did not reach statistical significance (*p*=0.079). Similarly, total motility was slightly higher in the UU-positive group (48.57%) than in the UU-negative group (46.16%), but there was no statistically significant difference (*p=*0.088). Leukocyte count and sperm morphology were consistent across both groups (*p*=0.323 and *p*=0.293, respectively). However, anti-sperm antibody (ASA) levels showed a statistically significant difference, with the UU-positive group having a higher median ASA percentage (*p=*0.020). Additionally, HDS, which reflects sperm nuclear immaturity, was notably elevated in the UU-positive group (median 6.54%) compared to the UU-negative group (median 5.75%) (*p=*0.014). Among the UU-positive group, DFI was slightly higher, but this difference was not statistically significant (*p=*0.276). In summary, although most semen parameters were similar, the UU-positive group exhibited higher ASA levels and HDS values (**Table 2**).

**Table 2:** Semen parameters of the two groups of male infertile patients.

Variables	Total (n=1215)	UU-negative (n=966)	UU-positive (n=249)	*p*
Abstinence time (days; median (IQR))	4.00 (3.00, 7.00)	4.00 (3.00, 7.00)	4.00 (3.00, 7.00)	0.744
Semen volume (ml; median (IQR))	3.00 (2.10, 4.00)	3.00 (2.10, 4.00)	3.00 (2.00, 4.00)	0.566
Sperm concentration (×10^6^/ml; median (IQR))	77.04 (46.38, 127.68)	78.61 (47.18, 127.32)	75.40 (43.89, 131.94)	0.706
Progressive motility (%; median (IQR))	39.63 (29.05, 51.52)	39.22 (27.96, 51.59)	41.77 (31.35, 51.38)	0.079
Total motility (%; median (IQR))	46.60 (34.36, 59.45)	46.16 (33.89, 59.25)	48.57 (36.69, 59.90)	0.088
Leukocyte count (×10^6^/ml; median (IQR))	0.16 (0.08, 0.35)	0.16 (0.08, 0.35)	0.16 (0.09, 0.35)	0.323
AsA (%; median (IQR))	2.00 (1.00, 4.00)	2.00 (1.00, 4.00)	2.00 (1.00, 5.00)	0.020
Normal morphology (%; median (IQR))	6.00 (4.00, 7.00)	6.00 (4.00, 7.00)	6.00 (4.00, 8.00)	0.293
DFI (%; median (IQR); n=139)	12.92 (9.34, 18.33)	12.79 (9.35, 18.18)	14.28 (9.00, 18.69)	0.276
HDS (%; median (IQR); n=139)	6.01 (4.66, 7.86)	5.75 (4.62, 7.68)	6.54 (5.14, 8.74)	0.014

IQR, interquartile range (25% and 75% percentiles); AsA, antisperm antibody; DFI, DNA fragmentation index; HDS, high DNA stainability.

### IVF embryo and pregnancy parameters

The comparison of embryo quality and pregnancy outcomes between UU-positive and UU-negative groups showed no significant differences in the normal fertilization rate (67.72% vs. 66.17%; *p=*0.133). The rate of high-quality embryos was comparable between the groups, with 54.49% in the UU-positive group and 55.13% in the UU-negative group (*p=*0.629). The blastocyst formation rate was also similar, at 48.87% for the UU-positive group and 49.05% for the UU-negative group (*p=*0.909). The embryo implantation rates were 38.69% for the UU-positive group and 39.73% for the UU-negative group (*p=*0.688). In the UU-positive group, the clinical pregnancy rate was 61.45%, compared to 63.56% in the UU-negative group (*p=*0.537). Although the miscarriage rate was higher in the UU-positive group (20.26%) than in the UU-negative group (14.01%), it was not statistically significant (*p=*0.054). The live birth rate was 48.99% in the UU-positive group and 54.66% in the UU-negative group (*p=*0.110) (**Table 3**).

**Table 3:** Embryo quality and pregnancy outcomes parameters.

Variables	Total (n=1215)	UU-negative (n=966)	UU-positive (n=249)	*p*
the number of oocytes (n (%))	11.19 ± 7.26	11.38 ± 7.33	10.44 ± 6.95	0.068
Normal fertilization rate, n (%)	9034 (66.47)	7274 (66.17)	1760 (67.72)	0.133
High-quality embryo rate, n (%)	4969 (55.01)	4010 (55.13)	959 (54.49)	0.629
Blastocyst formation rate, n (%)	2966 (49.02)	2383 (49.05)	583 (48.87)	0.909
Embryo implantation rate, n (%)	900 (39.53)	729 (39.73)	171 (38.69)	0.688
Clinical pregnancy rate, n (%)	767 (63.13)	614 (63.56)	153 (61.45)	0.537
Miscarriage rate, n (%)	117 (15.25)	86 (14.01)	31 (20.26)	0.054
Live birth rate, n (%)	650 (53.49)	528 (54.66)	122 (48.99)	0.110

### IVF neonatal outcomes

The average gestational age was 38.07 weeks in the UU-positive group and 38.31 weeks in the UU-negative group (*p=*0.291). The preterm delivery rate was slightly higher in the UU-positive group at 20.49% compared to 18.94% in the UU-negative group (*p=*0.695). The distribution of delivery types indicated that normal deliveries were more common in the UU-positive group (40.98%) than in the UU-negative group (33.33%), but this difference was not statistically significant (*p=*0.110). Both groups had nearly identical rates of singleton pregnancies, with 84.43% in the UU-positive group and 84.09% in the UU-negative group (*p=*0.927). The distribution of neonatal sex was consistent across both groups, with no significant difference (*p=*0.978). Apgar scores were also similar, with a mean of 9.83 in the UU-positive group and 9.81 in the UU-negative group (*p=*0.685). The newborns’ height and weight were similar across the groups, showing no notable discrepancies (*p=*0.879 and *p=*0.559, respectively). The incidence of very low birth weight and low birth weight was marginally elevated in the UU-positive group (2.13% vs.1.47%, *p=*0.478 and 19.15% vs.16.18%, *p=*0.394), but the differences were not statistically significant. Compared to the UU-negative group (4.08%), macrosomia occurrence was lower in the UU-positive group (1.41%), however, this difference did not reach statistical significance (*p=*0.125) (**Table 4**).

**Table 4:** Neonatal outcomes in this study.

Variables	Total (n=1215)	UU-negative (n=966)	UU-positive (n=249)	*p*
Gestational age (weeks; mean ± SD)	38.26 ± 2.20	38.31 ± 2.16	38.07 ± 2.38	0.291
Preterm delivery rate (n (%))	125 (19.23)	100 (18.94)	25 (20.49)	0.695
Type of delivery (n (%))				0.110
Normal delivery	226 (34.77)	176 (33.33)	50 (40.98)	
Cesarean delivery	424 (65.23)	352 (66.67)	72 (59.02)	
Singleton pregnancies rate, n (%)	547 (84.15)	444 (84.09)	103 (84.43)	0.927
Twin pregnancies rate, n (%)	103 (15.85)	84 (15.91)	19 (15.57)	
Neonatal sex rate (n (%))				0.978
Female neonate	341 (45.29)	277 (45.26)	64 (45.39)	
Male neonate	412 (54.71)	335 (54.74)	77 (54.61)	
Apgar score (mean ± SD)	9.81 ± 0.51	9.81 ± 0.52	9.83 ± 0.48	0.685
Neonatal weight (kg; mean ± SD)	3.07 ± 0.66	3.08 ± 0.66	3.04 ± 0.67	0.559
Neonatal height (cm; mean ± SD)	49.07 ± 2.64	49.07 ± 2.63	49.04 ± 2.71	0.878
Very low birth weight (< 1500 g) n (%)	12 (1.59)	9 (1.47)	3 (2.13)	0.478
Low birth weight (1500-2499 g) n (%)	126 (16.73)	99 (16.18)	27 (19.15)	0.394
Normal birth weight (2500-3999 g) n (%)	588 (78.09)	479 (78.27)	109 (77.31)	0.803
Macrosomia (≥ 4000 g) n (%)	27 (3.59)	25 (4.08)	2 (1.41)	0.125

IQR, interquartile range (25% and 75% percentiles); SD, standard deviation.

## Discussion

Approximately 15% of male infertility patients present with concurrent genital tract infections (13). Colonization by Mycoplasma in the urogenital tract is common, with a significant number of asymptomatic carriers, especially for UU in the male reproductive system (14, 15). However, there is ongoing debate regarding the effect of male reproductive system infection, particularly semen UU infections, on semen quality and pregnancy outcomes (16). Several studies have suggested that genital tract infections can lead to oligoasthenozoospermia as well as abnormal sperm DFI and HDS (6). However, other studies have found no apparent correlation between genital tract infection, male semen parameters, and adverse pregnancy outcomes (17).

This study investigates the impact of UU infection on various semen parameters, embryo quality, pregnancy outcomes, and neonatal outcomes within a cohort of 1,215 male patients, divided into UU-positive and UU-negative groups. The results indicate no significant differences in baseline characteristics between these groups, while male semen analysis revealed higher ASA levels and high DNA stainability (HDS) in the UU-positive group. In contrast, other semen parameters showed no significant differences. Similarly, the study found no substantial differences in IVF embryo quality, pregnancy outcomes, and neonatal outcomes between the UU-positive and UU-negative groups. These findings suggest that while UU infection may affect specific semen parameters, it does not significantly impact overall IVF reproductive outcomes.

The observed differences in semen parameters, particularly in ASA levels and HDS, are consistent with previous studies highlighting the potential pathogenic role of UU in male infertility (18) reported that UU and *Ureaplasma parvum* (UPA) infections could impair semen motility and induce inflammation, contributing to male infertility. The increase in ASA levels in the UU-positive group supports the hypothesis that UU infection can trigger an immune response, leading to the production of antibodies against sperm, which could impair sperm function and reduce fertility potential (18). Moreover, the higher HDS values observed in the UU-positive group align with findings by Aghazarian et al. (19), who demonstrated that UU infection is associated with increased DNA damage in sperm and reduced sperm quality. This suggests that UU may contribute to male infertility through mechanisms involving both immunological responses and direct damage to sperm DNA.

Despite the differences in ASA levels and HDS, the overall semen parameters, including sperm concentration, motility, and morphology, did not show significant differences between the UU-positive and UU-negative groups. Fu et al. (3) observed that although UU infection is associated with decreased motility and concentration of sperm, these effects might not be significant in all cases. The lack of significant differences in most semen parameters could be due to the variability in individual responses to UU infection, as well as differences in the severity and duration of infection. Furthermore, studies have shown that other factors, such as co-infections with other urogenital pathogens and individual genetic susceptibility, might influence the extent of the impact of UU on semen quality (20, 21). Although the UU-positive and UU-negative groups showed significant differences in HDS, the proportion of HDS in the UU-positive group remained under 15% threshold that is commonly thought to impact embryo quality (22). Additionally, studies conducted on mice and humans have revealed that oocytes might repair DNA damage (23).

The study also assessed the impact of UU infection on IVF outcomes, including embryo quality, pregnancy rates, and neonatal outcomes. The findings indicate no significant differences in these parameters between the UU-positive and UU-negative groups, suggesting that UU infection may not substantially affect the success of IVF treatments. This aligns with the findings of previous studies that have shown mixed or inconclusive evidence regarding the impact of UU on reproductive outcomes. For instance, Bai et al. (8), found no significant differences in IVF outcomes between men with and without UU infection, while other studies have reported slight reductions in pregnancy rates and increased miscarriage rates in UU-positive couples. The present study’s findings contribute to the growing body of evidence suggesting that while UU may impact semen quality, its effects on IVF outcomes may be less pronounced, particularly when other factors, such as the quality of oocytes and embryos, are optimal.

The neonatal outcomes examined in this study, including gestational age, preterm delivery rates, birth weight, and Apgar scores, also showed no significant differences between the UU-positive and UU-negative groups. This finding is consistent with the results of several studies that have reported no substantial impact of UU infection on neonatal health (24, 25). However, it is essential to note that while UU infection may not directly affect neonatal outcomes, the potential for long-term health effects on offspring due to subclinical infections or immune responses in the mother and fetus cannot be entirely ruled out (26). It is noteworthy that these microorganisms have the potential to cause a range of urogenital infections, including urethritis, prostatitis, epididymitis, and orchitis. Consequently, male patients testing positive for UU should be considered for eradication therapy with suitable antibiotic regimens. Further research is needed to explore the potential long-term consequences of UU infection during pregnancy and its implications for neonatal and child health.

Despite the comprehensive nature of this study, several limitations should be considered. First, the cross-sectional design limits the ability to establish causal relationships between UU infection and the observed outcomes. Longitudinal studies with larger sample sizes and follow-up data would provide more robust evidence regarding the impact of UU on male fertility and reproductive outcomes. Additionally, the culture method was unable to provide an absolute quantitative analysis of UU, limiting the ability to precisely measure its load in clinical samples. The amount of UU may be associated with disease outcomes. Therefore, more accurate quantification could improve our understanding of its clinical significance. Although common pathogens known to impact male fertility were excluded, some uncommon bacterial species were not ruled out. Additionally, the study did not account for potential confounding factors, such as the presence of other urogenital pathogens, the duration and severity of UU infection, and the patients’ previous treatment history, which could influence the results. Finally, the reliance on standard semen parameters may not capture the full extent of the impact of UU on sperm function, and more advanced diagnostic techniques, such as proteomics and genomics, could provide deeper insights into the molecular mechanisms underlying UU-related infertility.

In summary, our study’s results suggest that male UU infection may have some adverse effects on semen parameters. but IVF technology remains a viable and safe option for infertile couples with male UU infection.

## Data Availability

The raw data supporting the conclusions of this article will be made available by the authors, without undue reservation.

## References

[1] Zanotta N Campisciano G Morassut S Castro-Silva E Luksa V Zito G. Emerging role for Ureaplasma parvum serovar 3: Active infection in women with silent high-risk human papillomavirus and in women with idiopathic infertility. J Cell Physiol. (2019) 234:17905–11. doi: 10.1002/jcp.v234.10 30883747

[2] Bai S Li Y Wan Y Guo T Jin Q Liu R. Sexually transmitted infections and semen quality from subfertile men with and without leukocytospermia. Reprod Biol endocrinology: RB&E. (2021) 19:92. doi: 10.1186/s12958-021-00769-2 34154600 PMC8218447

[3] Xianchun F Jun F Zhijun D Mingyun H. Effects of Ureaplasma urealyticum infection on semen quality and sperm morphology. Front Endocrinol. (2023) 14:1113130. doi: 10.3389/fendo.2023.1113130 PMC10025488 36950686

[4] Zeng J Wu T Wang L Yu L Lin H Chen Z. Characteristics of reproductive tract infections caused by common pathogens among the outpatients of reproductive medicine center in Putian: retrospective study. BMC Infect Dis. (2024) 24:315. doi: 10.1186/s12879-024-09180-9 38486167 PMC10941379

[5] Tantengco OAG de Castro Silva M Velayo CL. The role of genital mycoplasma infection in female infertility: A systematic review and meta-analysis. Am J Reprod Immunol (New York NY: 1989). (2021) 85:e13390. doi: 10.1111/aji.13390 33417733

[6] Zhang QF Zhang YJ Wang S Wei Y Li F Feng KJ. The effect of screening and treatment of Ureaplasma urealyticum infection on semen parameters in asymptomatic leukocytospermia: a case-control study. BMC Urology. (2020) 20:165. doi: 10.1186/s12894-020-00742-y 33092572 PMC7579809

[7] Liu KS Mao XD Pan F An RF. Effect and mechanisms of reproductive tract infection on oxidative stress parameters, sperm DNA fragmentation, and semen quality in infertile males. Reprod Biol endocrinology: RB&E. (2021) 19:97. doi: 10.1186/s12958-021-00781-6 34183027 PMC8237428

[8] Bai S Li Y Hu MH Wu L Shui LJ Wang XH. Association of sexually transmitted infection with semen quality in men from couples with primary and secondary infertility. Asian J Androl. (2022) 24:317–22. doi: 10.4103/aja202164 PMC9226702 34782548

[9] Liu H Song X Huang M Zhan H Wang S Zhu S. Ureaplasma urealyticum induces polymorphonuclear elastase to change semen properties and reduce sperm motility: a prospective observational study. J Int Med Res. (2022) 50:3000605221106410. doi: 10.1177/03000605221106410 35701892 PMC9208062

[10] Huang C Long X Jing S Fan L Xu K Wang S. Ureaplasma urealyticum and Mycoplasma hominis infections and semen quality in 19,098 infertile men in China. World J Urology. (2016) 34:1039–44. doi: 10.1007/s00345-015-1724-z 26537883

[11] Zhou YH Ma HX Shi XX Liu Y. Ureaplasma spp. in male infertility and its relationship with semen quality and seminal plasma components. J microbiology immunology infection = Wei mian yu gan ran za zhi. (2018) 51:778–83. doi: 10.1016/j.jmii.2016.09.004 28739435

[12] Evenson DP Djira G Kasperson K Christianson J. Relationships between the age of 25,445 men attending infertility clinics and sperm chromatin structure assay (SCSA(R)) defined sperm DNA and chromatin integrity. Fertil Steril. (2020) 114:311–20. doi: 10.1016/j.fertnstert.2020.03.028 32653083

[13] Rivero MJ Kulkarni N Thirumavalavan N Ramasamy R. Evaluation and management of male genital tract infections in the setting of male infertility: an updated review. Curr Opin Urol. (2023) 33:180–6. doi: 10.1097/MOU.0000000000001081 PMC10073322 36861760

[14] Liu J Wang Q Ji X Guo S Dai Y Zhang Z. Prevalence of Ureaplasma urealyticum, Mycoplasma hominis, Chlamydia trachomatis infections, and semen quality in infertile and fertile men in China. Urology. (2014) 83:795–9. doi: 10.1016/j.urology.2013.11.009 24411218

[15] Ozturk S Yildiz S Dursun P Yener Ilce B Kaymaz O. Mycoplasma hominis profile in women: Culture, kit, molecular diagnosis, antimicrobial resistance, and treatment. Microb Pathog. (2019) 135:103635. doi: 10.1016/j.micpath.2019.103635 31352064

[16] Beeton ML Payne MS Jones L. The Role of Ureaplasma spp. in the Development of Nongonococcal Urethritis and Infertility among Men. Clin Microbiol Rev. (2019) 32:e00137-18. doi: 10.1128/CMR.00137-18 31270127 PMC6750135

[17] Ricci S De Giorgi S Lazzeri E Luddi A Rossi S Piomboni P. Impact of asymptomatic genital tract infections on *in vitro* Fertilization (IVF) outcome. PloS One. (2018) 13:e0207684. doi: 10.1371/journal.pone.0207684 30444931 PMC6239332

[18] Zhou Y Wu X Shen B Ma H. The relationship between ureaplasma species and male infertility: pathogenicity, biology, diagnosis, and treatment. Altern therapies Health Med. (2024) 30:96–102. 38743894

[19] Aghazarian A Huf W Klingler HC Klatte T. The effect of seminal pathogens on standard semen parameters, sperm kinematics and seminal inflammatory markers. J Reprod Immunol. (2024) 161:104183. doi: 10.1016/j.jri.2023.104183 38154434

[20] Paira DA Olivera C Tissera AD Molina RI Olmedo JJ Rivero VE. Ureaplasma urealyticum and Mycoplasma hominis urogenital infections associate with semen inflammation and decreased sperm quality. J Leukocyte Biol. (2023) 113:18–26. doi: 10.1093/jleuko/qiac006 36822158

[21] Sharma R Gupta S Agarwal A Henkel R Finelli R Parekh N. Relevance of leukocytospermia and semen culture and its true place in diagnosing and treating male infertility. World J Men’s Health. (2022) 40:191–207. doi: 10.5534/wjmh.210063 34169683 PMC8987138

[22] Luo Y Wu S Zhang M Zhou H Yuan J Yang Y. Sperm DNA integrity is critically impacted by male age but does not influence outcomes of artificial insemination by husband in the Chinese infertile couples. Aging (Albany NY). (2022) 14:4326–35. doi: 10.18632/aging.204058 PMC9186781 35580171

[23] Sun TC Zhang Y Li HT Liu XM Yi DX Tian L. Sperm DNA fragmentation index, as measured by sperm chromatin dispersion, might not predict assisted reproductive outcome. Taiwan J Obstet Gynecol. (2018) 57:493–8. doi: 10.1016/j.tjog.2018.06.003 30122567

[24] Dehghan A Pourmand MR Salimi V Asbagh FA Foroushani AR Sadeghi K. The effects of Chlamydia trachomatis, Mycoplasma hominis, and Ureaplasma urealyticum loads on semen quality: Detection and quantitative analysis. Microb Pathog. (2022) 169:105676. doi: 10.1016/j.micpath.2022.105676 35820579

[25] Yasynetskyi M Banyra O Nikitin O Ventskivska I Kozlov V Kvach M. Mixed sexually transmitted infections in infertile couples: empirical treatment and influence on semen quality. Recent Adv Anti-Infect Drug Discov. (2021) 16:227–36. doi: 10.2174/2772434416666211129105145 34844551

[26] Tam Le M Nguyen Nguyen D Bach Nguyen H Quynh Tram Ngo V Quoc Huy Nguyen V. Ureaplasma urealyticum and Mycoplasma genitalium detection and sperm quality: A cross-sectional study in Vietnam. Int J Reprod Biomed. (2021) 20:185–94. doi: 10.18502/ijrm.v20i3.10710 PMC9099368 35571499

